# Structural perspectives on clinical β-lactamase inhibitors: From mechanism to resistance

**DOI:** 10.71150/jm.2510019

**Published:** 2026-03-19

**Authors:** Soo-Bong Park, Myeong-Yeon Kim, Sun-Shin Cha

**Affiliations:** 1 Department of Chemistry and Nanoscience, Ewha Womans University, Seoul 03760, Republic of Korea; 2 Graduate Program in Innovative Biomaterials Convergence, Ewha Womans University, Seoul 03760, Republic of Korea; 3 R&D Division, TODD PHARM CO. LTD., Seoul 03760, Republic of Korea

**Keywords:** structure and function of β-lactamase inhibitor (BLI), inhibition mechanism, inhibition spectrum, clinically isolated β-lactamase resistance against BLI, pan-β-lactamase inhibitor, BLI with novel scaffold

## Abstract

β-Lactam antibiotics marked the beginning of an era of effective and safe treatment for bacterial infections and remain the most widely prescribed antibacterial agents today. However, the emergence of antibiotic-resistant bacteria threatens a return to the pre-antibiotic era. In particular, bacterial expression of β-lactamases inactivating β-lactam antibiotics presents a challenge in antimicrobial therapy. While inhibitors against β-lactamases have been developed to protect the therapeutic efficacy of β-lactam antibiotics, the clinical use of β-lactamase inhibitors is constrained due to their limited inhibition spectrum and the emergence of inhibitor-resistant β-lactamase variants. As an effort to tackle this issue, here we reviewed the structural and mechanistic features of β-lactamases and their FDA-approved inhibitors. Moreover, mutations in clinically isolated β-lactamases that confer resistance against their inhibitors are compiled. The comprehensive overview offered in this review aims to support and stimulate the design of next-generation β-lactamase inhibitors for combating β-lactamase-mediated antibiotic resistance.

## Introduction

**Fig. 1.**
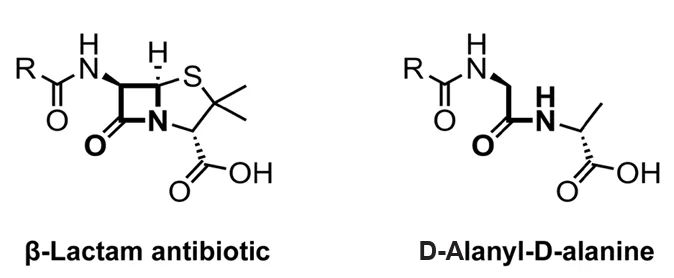
The structure of β-lactam antibiotic and the D-alanyl-D-alanine dipeptide. The β-lactam ring and peptide bonds involved in the mimicry are shown in bold lines.

β-Lactam antibiotics are the most widely used class of antibacterial agents in clinical practice, accounting for 54.8% of total global antibiotic consumption (Bush and Bradford, 2016; Klein et al., 2024). They have a four-membered β-lactam ring that mimics the D-alanyl-D-alanine (Fig. 1), the natural substrate of penicillin-binding proteins (PBPs), which are enzymes crucial for bacterial cell wall synthesis (Kim et al., 2023; Yocum et al., 1979). The β-lactam ring allows β-lactam antibiotics to bind covalently to PBPs, thereby inhibiting their enzyme activity, weakening the bacterial cell wall, and ultimately causing bacterial cell death (Mora-Ochomogo and Lohans, 2021; Tipper and Strominger, 1965). The antibacterial activity of β-lactam antibiotics critically depends on the intact β-lactam ring. Additionally, modifications at the side chain diversify their antibacterial spectrum and stability, giving rise to the four major subclasses: penicillins, cephalosporins, monobactams, and carbapenems. Notably, carbapenems are regarded as last-resort antibiotics due to their broad spectrum and effectiveness against multidrug-resistant bacteria.

The predominant mechanism of β-lactam resistance in Gram-negative bacteria, which represent one of the most critical threats to global public health due to their ability to cause severe infections, is the production of β-lactamases that hydrolyze the β-lactam ring and abolish antibiotic activity (Bush and Bradford, 2020; De Oliveira et al., 2020; Zha et al., 2025). Based on amino acid sequence homology, β-lactamases are classified into four Ambler classes: A, B, C, and D (Ambler, 1980; Naas et al., 2017). These classes are further distinguished by their catalytic mechanisms: serine β-lactamases (SBLs; classes A, C, and D) utilize an active-site serine residue for β-lactam hydrolysis, while zinc-dependent metallo-β-lactamases (MBLs; class B) require one or two zinc ions for their enzymatic activity (Bush and Jacoby, 2010; Tooke et al., 2019a). To counteract these enzymes, β-lactamase inhibitors (BLIs) are co-administered with β-lactam antibiotics to preserve the antibacterial efficacy of their partner drugs (Bush and Bradford, 2016; Drawz and Bonomo, 2010; Reading and Cole, 1977; Tehrani and Martin, 2018). However, currently FDA-approved BLIs are ineffective against MBLs, notably the clinically significant carbapenemases VIM, IMP, and NDM, that hydrolyze last-resort carbapenems (Table 1, Meletis, 2016). The emergence of inhibitor-resistant (IR) variants further limits their spectrum of activity. These limitations underscore the urgent need for novel and broad-spectrum BLIs.

This review aims to (i) highlight the mechanisms and structural features of β-lactamase, (ii) structurally explain the inhibitory mechanisms and limitations of eight FDA-approved BLIs, and (iii) discuss resistance mechanisms of clinically isolated BLI-resistant β-lactamases. Through this review, we want to support the design of improved and innovative BLIs that can effectively address β-lactamase–driven antimicrobial resistance.

## Mechanistic and Structural Diversity of β-lactamases

### Differences in catalytic mechanisms between SBLs and MBLs

**Fig. 2.**
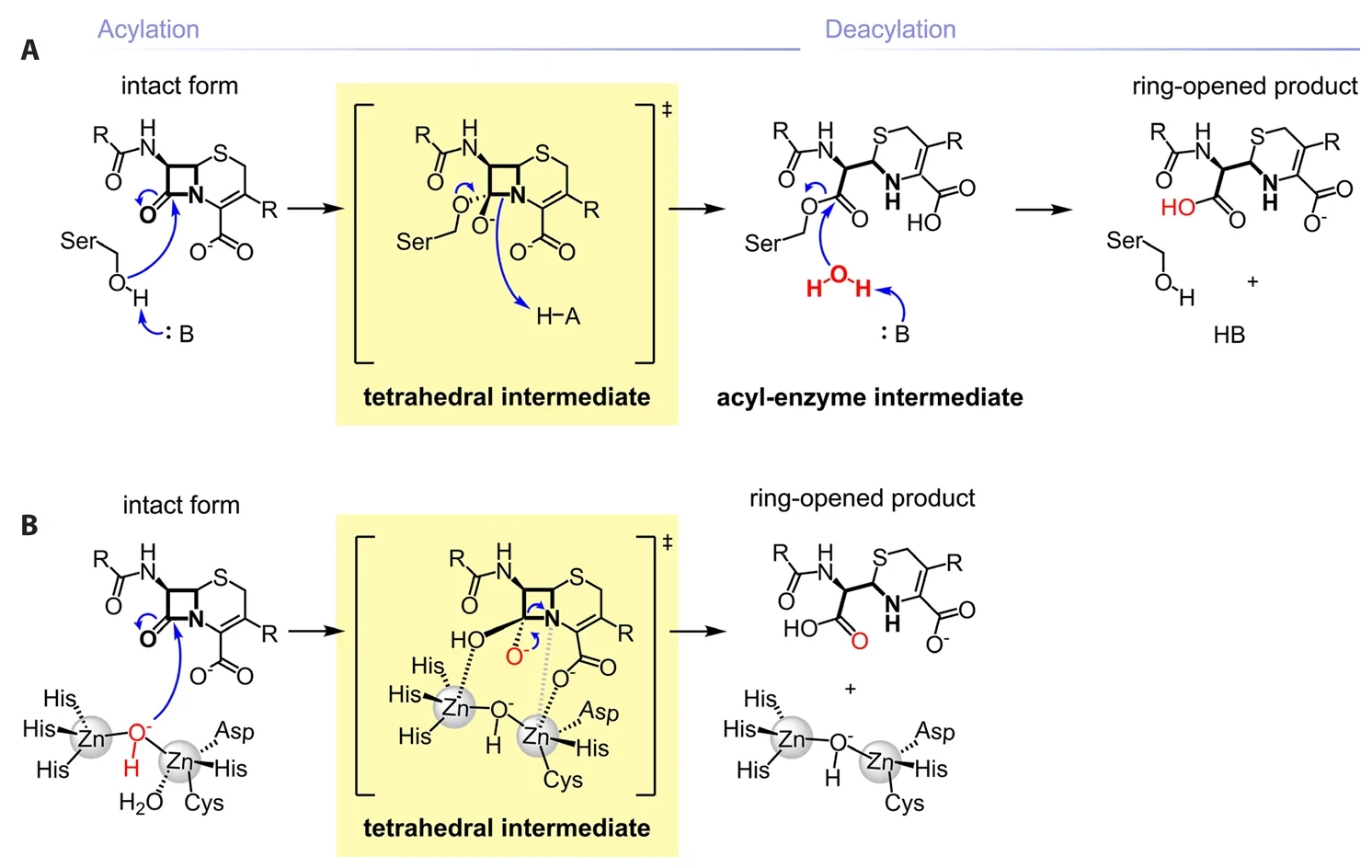
Distinct catalytic mechanisms of SBLs and MBLs. The reaction mechanisms for the hydrolysis of a β-lactam substrate (cephalosporin) by (A) SBLs and (B) MBLs. Blue arrows indicate the direction of electron flow. In the schematic, A and B denote the general acid and general base, respectively, that participate in proton transfer during catalysis. The yellow boxed region highlights the common tetrahedral intermediate shared by both SBLs and MBLs.

**Fig. 3.**
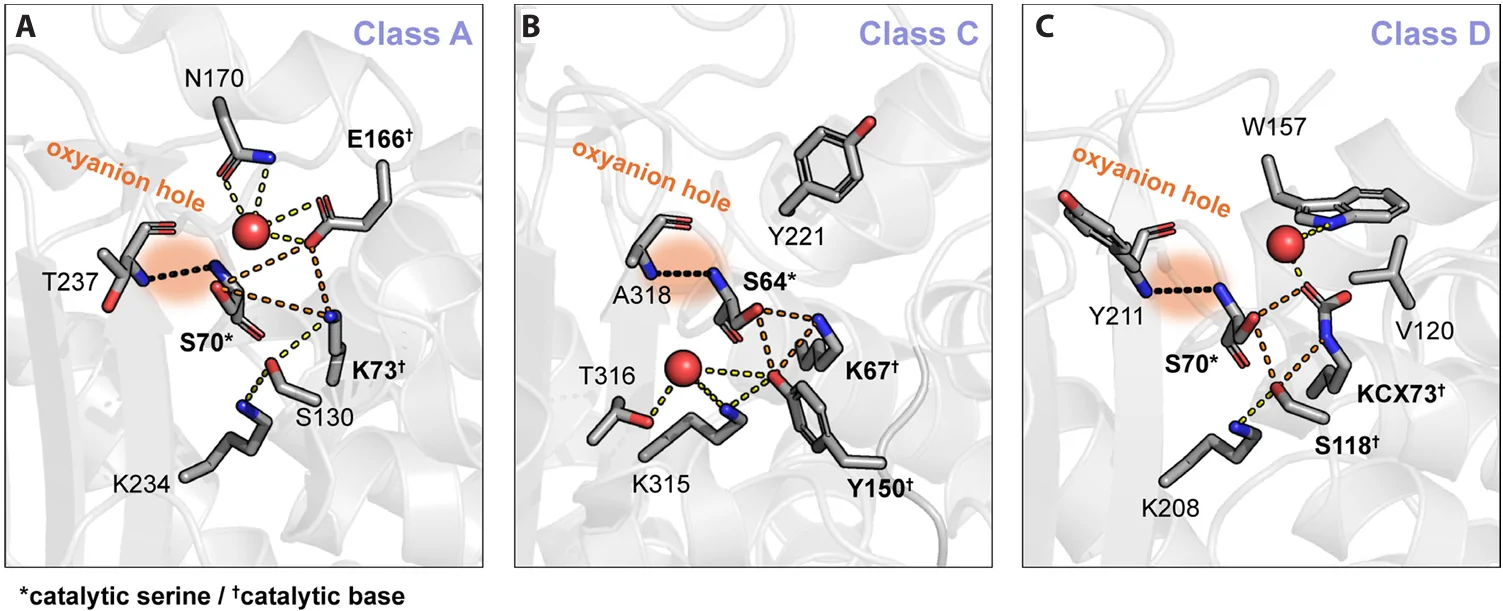
Key catalytic residues and hydrogen bond networks in class A, class C, and class D SBLs. Active sites of (A) KPC-2 (PDB 2OV5), (B) AmpC EC-1 (PDB 1KE4), and (C) OXA-48 (PDB 4S2K) are shown. The red sphere represents the deacylation water molecule. All catalytic residues are shown as sticks, while the two residues forming the oxyanion hole are highlighted in orange and displayed with both side- and main-chain atoms. Hydrogen bond networks among the catalytic residues are shown as dashed lines in three colors. Orange indicates the hydrogen bond between the catalytic serine and the general bases. Yellow represents the hydrogen bonds maintaining catalytic geometry. Black denotes the backbone hydrogen bonds forming the oxyanion hole. For class D, to visualize the deacylation water molecule, the structure was derived by removing Avibactam from the OXA-48/Avibactam complex (PDB 4S2K). In OXA-48, Ser70 is the equivalent residue to Ser67 in class D β-lactamases.

The mechanisms of β-lactamases for the hydrolysis of the β-lactam ring are fundamentally distinct in SBLs and MBLs (Fig. 2, Tooke et al., 2019a). SBLs catalyze β-lactam hydrolysis through a two-step process: during acylation, a conserved nucleophilic serine attacks the β-lactam carbonyl carbon to generate a tetrahedral intermediate that collapses to an acyl-enzyme intermediate upon β-lactam ring opening; this is followed by deacylation, in which an activated water molecule (deacylation water) hydrolyzes the acyl–enzyme bond to regenerate the free enzyme and release the ring-opened product (Fig. 2A, Gonzalez-Bello et al., 2020; Li et al., 2017). This process is supported by conserved general acid/base residues that not only facilitate serine activation but also activate and correctly position the deacylation water molecule. Additionally, the transition state is stabilized within the oxyanion hole (Fig. 3). In contrast, MBLs catalyze β-lactam hydrolysis via a single-step mechanism in which a metal-activated water molecule directly attacks the β-lactam carbonyl carbon, forming a tetrahedral intermediate that collapses to open the β-lactam ring and immediately release the product, without forming an acyl-enzyme intermediate (Fig. 2B, Gonzalez-Bello et al., 2020; Li et al., 2017). This mechanistic distinction between SBLs and MBLs, together with fundamental differences in their overall morphology, accounts for the current absence of clinically effective BLIs against MBLs, as these inhibitors were primarily developed to target SBLs (Mojica et al., 2022). Among SBLs, however, the inhibition efficiency and spectrum of BLIs are different across classes due to variability in their active-site architectures, despite the conservation of an overall fold (Tehrani and Martin, 2018).

### Diversity of active-site architectures across SBL classes

**Fig. 4.**
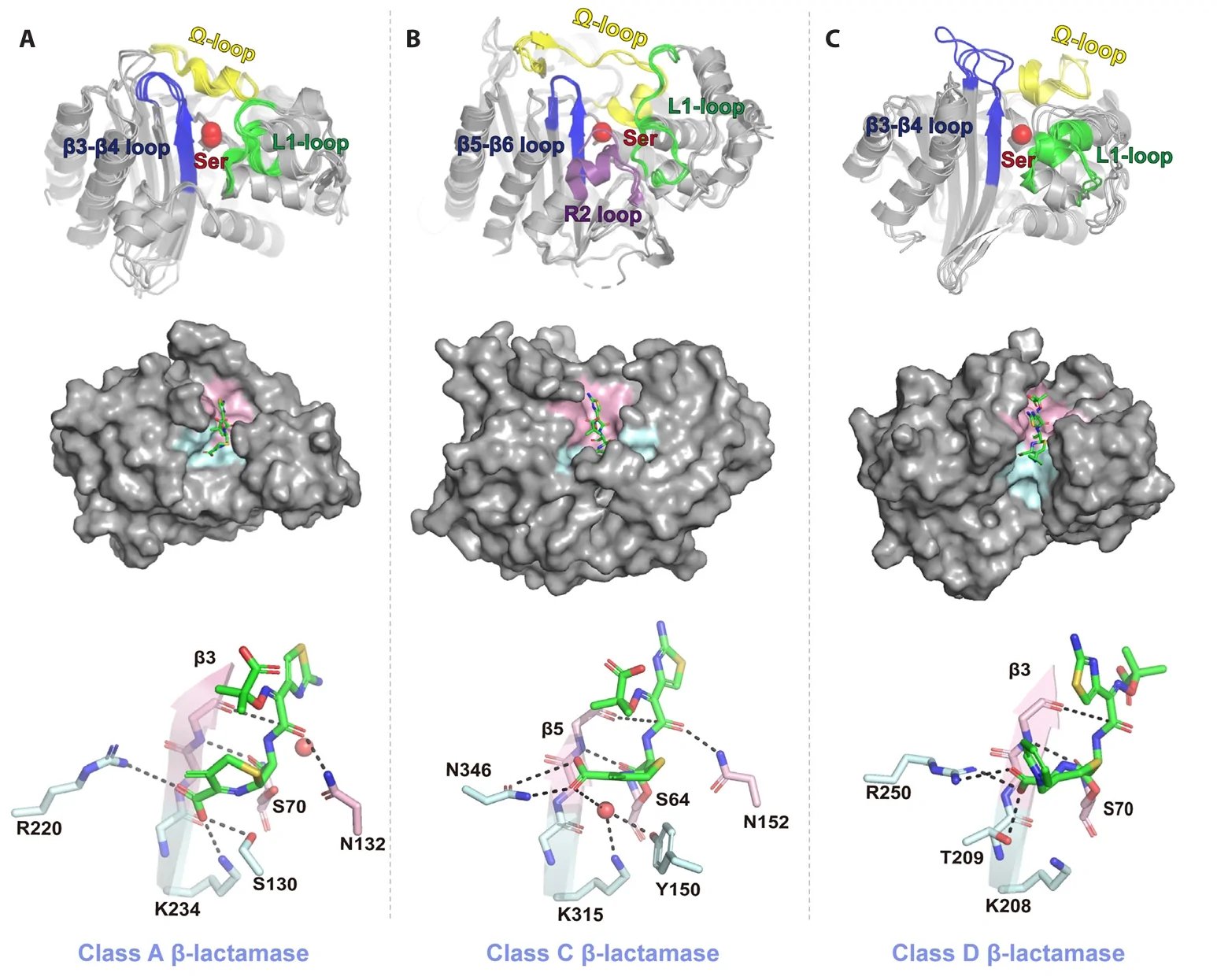
The active site and substrate-binding pocket architectures among class A, C, and D SBLs. Top panels show aligned representative enzymes of each class — (A) TEM-1 (PDB 7U6Q), SHV-1 (PDB 4FH4), CTX-M-14 (PDB 1YLT), and KPC-2 (PDB 2OV5); (B) AmpC EC-1 (PDB 1KE4), CMY-10 (PDB 1ZKJ), and ACC-1 (PDB 6K8X); (C) OXA-10 (PDB 1FOF), OXA-23 (PDB 4K0X), and OXA-48 (PDB 6P96). The catalytic serine residue is shown as a red sphere. Structural loops surrounding the active site are colored as follows: Ω-loop (yellow), β3–β4 or β5–β6 loop (blue), L1 loop (green), and R2 loop (purple; class C only). Middle and bottom panels display the protein surface and the ligand-binding mode of (A) KPC-2/ceftazidime complex (PDB 6Z24), (B) AmpC EC-1/ceftazidime complex (PDB 1IEL), and (C) OXA-48/ceftazidime complex (PDB 6Q5F), respectively. Ligands bound to the active site are shown as green sticks. The amide-binding subsite is highlighted in pink and the carboxylate-binding subsite in blue. Residues participating in ligand binding are shown as sticks, and hydrogen bonds are represented as black dashed lines.

**Fig. 5.**
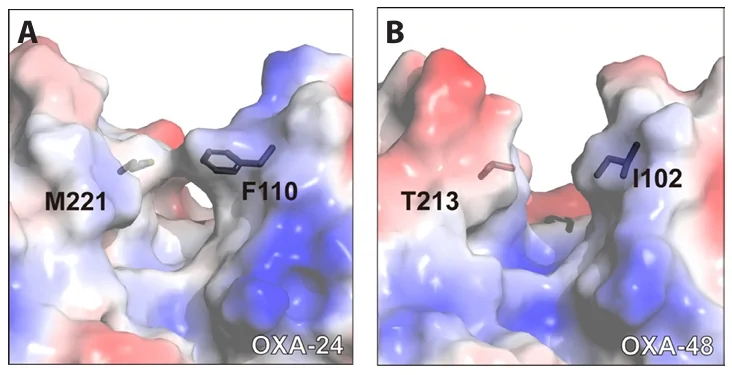
Surface electrostatic potential of the class D β-lactamase active sites. The surfaces are colored in red and blue for negative and positive charges, respectively. (A) Active-site surface of OXA-24 (PDB 4JF6) with the side chains of residues Phe110 and Met221, which form a hydrophobic bridge, shown as sticks. (B) Active-site surface of OXA-48 (PDB 6P96) with the side chains of the corresponding residues Ile102 and Thr213 shown as sticks.

The active sites of SBLs are defined by three loops (L1-loop, Ω-loop, and β3(5)-β4(6) loop) that surround the catalytic serine (Fig. 4), which corresponds to Ser70 in class A, Ser64 in class C, and Ser67 (and its equivalent residue) in class D β-lactamases (Fig. 3, Ambler et al., 1991; Hall and Barlow, 2003). They contain an oxyanion hole, amide-binding subsite, and carboxylate-binding subsite to recognize and stabilize the carbonyl oxygen, amide bond, and carboxylate motifs, respectively, found in most substrates and inhibitors (Figs. 3 and 4). Class A β-lactamases possess three relatively short loops forming an open-bottom, solvent-exposed pocket (Fig. 4A). Class C β-lactamases, in contrast, contain an additional R2-loop that encloses the bottom region of the active site (Fig. 4B). The R2- and Ω-loops of class C β-lactamases are relatively long and flexible (Lee et al., 2025), which facilitates either specific interactions or conformational constraints with ligands, thereby enhancing or restricting their accommodation within the catalytic pocket (Jeong et al., 2021; Kim et al., 2006; Lee et al., 2009; Na and Cha, 2016). Class D β-lactamases are characterized by an elongated β3–β4 loop that forms a narrow and hydrophobic pocket, rendering the pocket unfavorable for binding of polar or bulky ligands (Fig. 4C, Lee et al., 2025). Even certain class D β-lactamases, such as the OXA-23-like and OXA-24/40-like subfamilies, possess a distinct hydrophobic bridge that creates a narrower and more hydrophobic tunnel-like active site (Fig. 5, Stewart et al., 2019, 2022; Wang et al., 2021). Unlike classes A and C which employ Glu166 and Tyr150 as their general base, respectively, class D β-lactamases employ a carboxylated lysine (KCX, Fig. 3C) as the general acid/base, whose decarboxylation leads to enzyme inactivation. Accordingly, the hydrophobic active site of the class D β-lactamase contributes to maintaining enzyme activity by limiting water access, even for the deacylation water, thereby preserving the KCX from hydrolysis in aqueous environments (Toth et al., 2017).

## Structural Overview of Mechanisms and Limitations in FDA-Approved BLIs

### Overcoming β-lactamase–mediated resistance

**Fig. 6.**
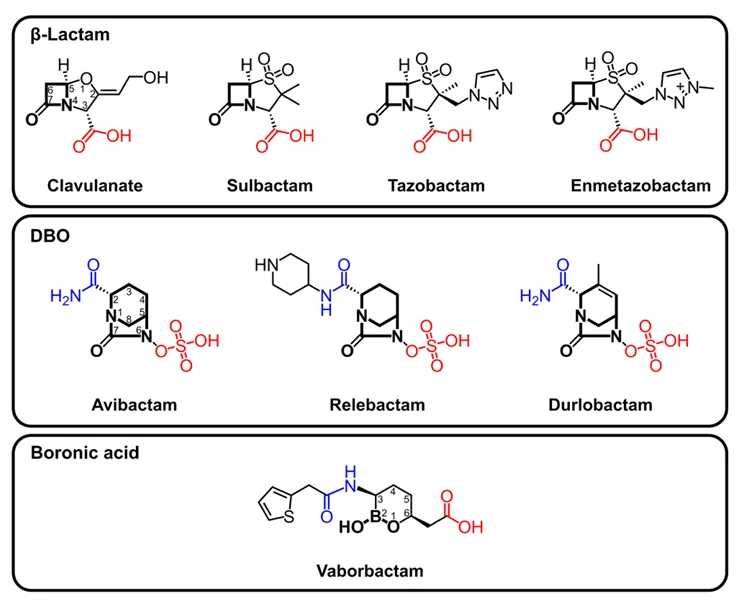
Overview of FDA-approved BLI scaffolds. The core structures—β-lactam, diazabicyclooctane (DBO), and boronic acid—are indicated in bold, the amide bond is highlighted in blue, and the carboxylate and sulfate groups are shown in red. Atom numbering for the rings follows the scheme provided on the left chemical.

Although β-lactamase–mediated antimicrobial resistance has driven the modifications of diverse β-lactam antibiotics, these efforts have been insufficient to overcome more than 2,000 of the β-lactamases and their variants (Bush, 2018). In response, BLIs were developed to preserve efficacy of β-lactam antibiotics by inactivating β-lactamase enzymatic activity. To date, eight BLIs have received FDA approval for co-administration with β-lactam antibiotics. They can be structurally classified into three major groups based on their reactive center: β-lactams (Clavulanate, Sulbactam, Tazobactam, and Enmetazobactam), diazabicyclooctanes (DBOs; Avibactam, Relebactam, and Durlobactam), and boronic acids (Vaborbactam, Fig. 6, Table 1). Here, we focus on the mechanisms of BLIs against SBLs according to their core chemical scaffolds, as these inhibitors show no activity against MBLs.

### Shared inhibition mechanisms of FDA-approved BLIs

**Fig. 7.**
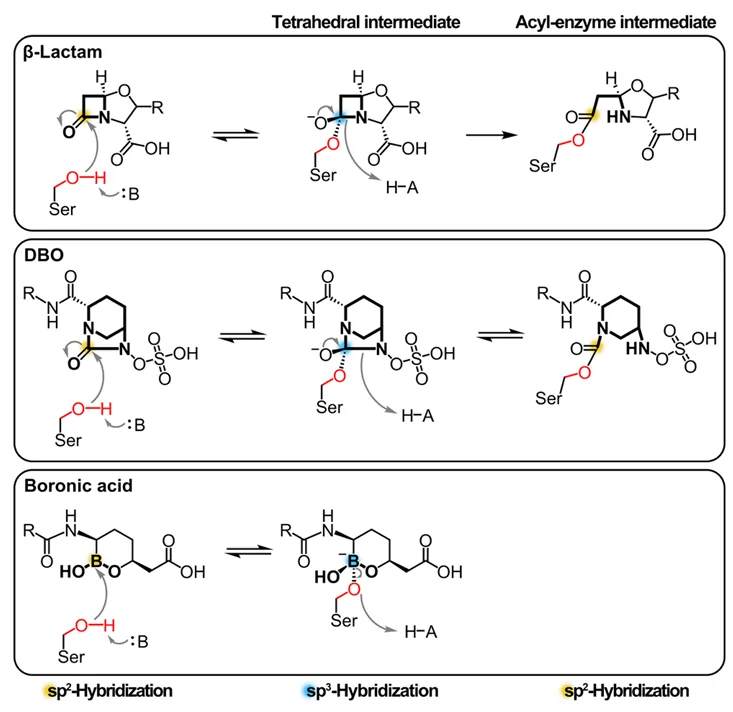
Acylation of BLIs by SBLs: structural analogues and transition state geometries. The core structures—β-lactam, diazabicyclooctane (DBO), and boronic acid—are indicated in bold and the electrophilic reaction centers are highlighted. Electron flow is indicated with gray arrows, and the side chain of serine is shown in red. The tetrahedral intermediate and acyl-enzyme intermediate analogs formed during acylation are arranged in the same column.

FDA-approved BLIs share a common inhibition strategy by mimicking the β-lactam hydrolysis mechanism of SBLs (Lang et al., 2022; Mora-Ochomogo and Lohans, 2021; van den Akker and Bonomo, 2018). Specifically, all inhibitors possess an electrophilic atom capable of forming a covalent bond with the catalytic serine. In β-lactam- and DBO-based BLIs, the carbonyl carbon (C7) serves as the electrophile (Fig. 6). Its partial positive charge arises from the electronegativity of adjacent oxygen and nitrogen atoms, combined with the ring strain. In boronic acid-based BLIs, the boron atom intrinsically functions as the electrophilic center due to its electron deficiency from a vacant *p*-orbital (Figs. 6 and 7, van den Akker and Bonomo, 2018).

Upon nucleophilic attack at their electrophilic center, β-lactam- and DBO-based BLIs undergo ring opening, thereby resembling the covalent acyl-enzyme intermediate formed during β-lactam substrate hydrolysis. In contrast, boronic acid-based BLIs mimic the tetrahedral intermediate in the enzymatic pathway without ring opening (Fig. 7, van den Akker and Bonomo, 2018). These intermediate analogs are thermodynamically stable due to their chemical structures, interactions within the enzyme active site, or both. This thermodynamic favorability is reflected in the increased activation energy barrier for deacylation, energetically disfavoring further chemical transformation. Some intermediates also inhibit the access or activation of the deacylation water. Through one or both of the mechanisms described above, BLIs exhibit exceptionally low turnover rates, which confer high kinetic stability. Consequently, the inhibitors resist hydrolytic degradation, preventing enzyme regeneration and maintaining prolonged inhibition (Chen and Herzberg, 1992; Hecker et al., 2015; Kalp et al., 2009; Lahiri et al., 2014b; Lang et al., 2024; Papp-Wallace et al., 2023; Tsivkovski and Lomovskaya, 2020; van den Akker and Bonomo, 2018).

## β-Lactam-Based BLIs

### β-Lactam-based inhibitors: Clavulanate to Enmetazobactam

In 1984, the first β-lactamase inhibitor, Clavulanate, received FDA approval, marking a new era in combating antibiotic resistance through enzyme inhibition. Clavulanate, a natural product possessing a β-lactam ring, is resistant to hydrolysis by some β-lactamases and inhibits their enzymatic activity (Reading and Cole, 1977). This discovery led to the development of various β-lactam-based inhibitors, and currently, four inhibitors—Clavulanic acid, Sulbactam, Tazobactam, and Enmetazobactam—have FDA approval for co-administration with β-lactam antibiotics (Table 1). Structurally, they share a β-lactam ring fused to a five-membered ring that includes a carboxyl group (Fig. 6). Based on the type of ring fused to the β-lactam core, these inhibitors are classified into two structural groups: Clavulanate with an oxazolidine ring fusion (penam) and Sulbactam, Tazobactam, and Enmetazobactam with thiazolidine ring fusion (penam sulfone). These inhibitors also differ in their substituents at the C2 position: Clavulanate contains a 2-hydroxyethylidene group, Sulbactam has two methyl groups (gem-dimethyl), Tazobactam possesses a 1,2,3-triazole ring, and Enmetazobactam features a methylated triazole ring (Fig. 6).

### β-Lactam-based suicide inhibitors acting via second ring opening to a stable trans-enamine intermediate

β-Lactam-based inhibitors, commonly known as “suicide inhibitors,” irreversibly inhibit some class A β-lactamases such as TEM-1 and SHV-1 (Bush et al., 1993), but they have limitations in inhibiting extended-spectrum class A enzymes, class B, class C, or most class D β-lactamases (Table 1, Lang et al., 2024; Mojica et al., 2022; Papp-Wallace et al., 2012; Totir et al., 2008).

**Fig. 8.**
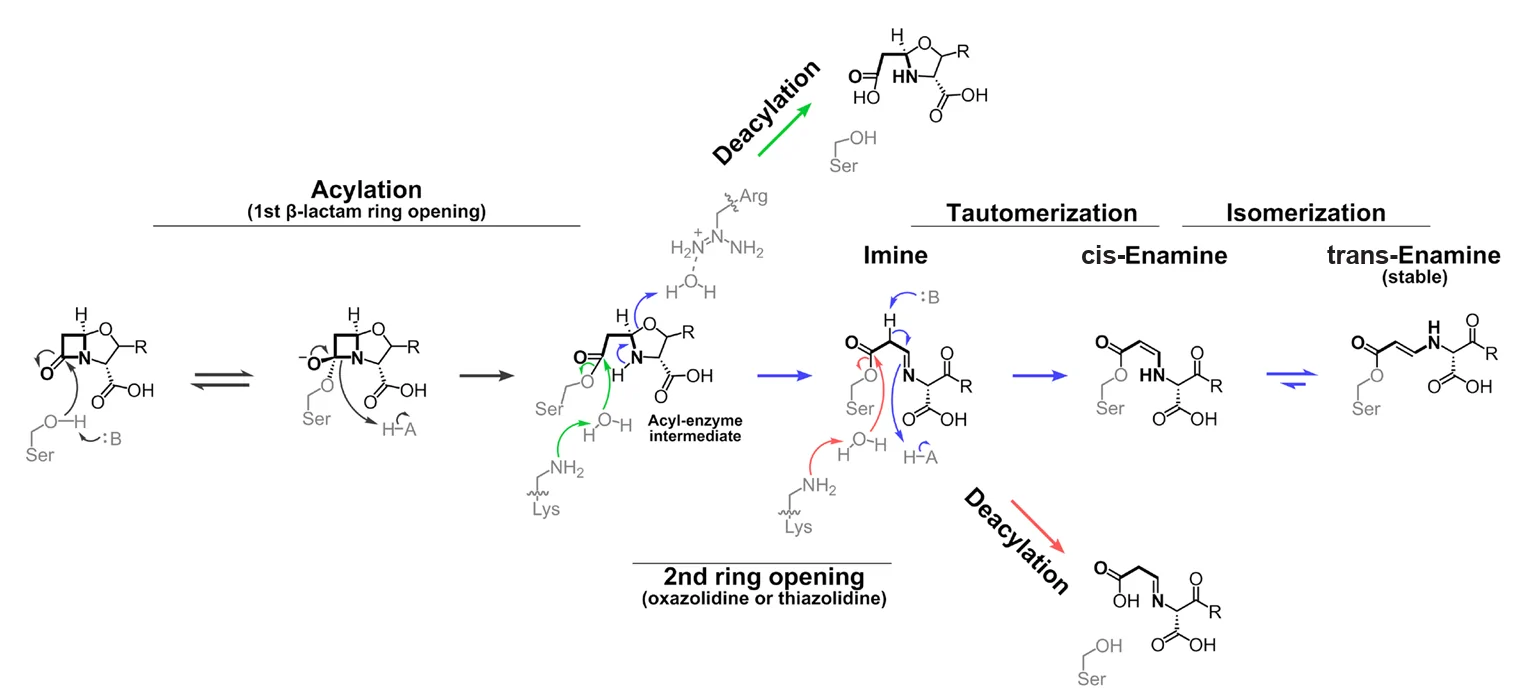
Mechanistic pathway of β-lactam-based inhibitor with SBLs. After acyl-enzyme complex formation, the second ring opening leads to either release of the inactive degraded inhibitor via deacylation (green arrows) or structural conversion to imine and enamine forms (blue arrows). If the imine does not efficiently convert to enamine, it is also released as an inactive product through the deacylation pathway (red arrows).

The initial binding of β-lactam inhibitors to class A β-lactamases is facilitated by the carboxylate-binding subsite, formed by arginine (Arg244 in TEM-1 and Arg243 in TEM-171) and conserved Lys234, which recognizes the carboxyl group and properly orients the β-lactam ring for efficient acylation (Fig. 15B, Mehta et al., 2021; Thomson et al., 2006; Tondi et al., 2014). After initial binding, the active-site serine performs a nucleophilic attack on the carbonyl carbon (C7), forming a covalent tetrahedral intermediate that subsequently collapses with opening of the β-lactam ring to yield the acyl-enzyme intermediate (Fig. 8). Early mechanistic models proposed that such acylated inhibitors underwent fragmentation into aldehydes, leading to irreversible inhibition (Brown et al., 1996; Power et al., 2012; Ruggiero et al., 2014). However, recent work has demonstrated that this fragmentation is an analytical artifact caused by the acid used as a mobile-phase additive in mass spectrometry. Under neutral conditions, mass spectrometry, ^1^H NMR spectroscopy, quantum mechanics/molecular mechanics calculations, and X-ray crystallography have shown that a trans-enamine intermediate is responsible for irreversible inhibition activity of class A β-lactamases (Lang et al., 2022, 2024; Li et al., 2011).

**Fig. 9.**
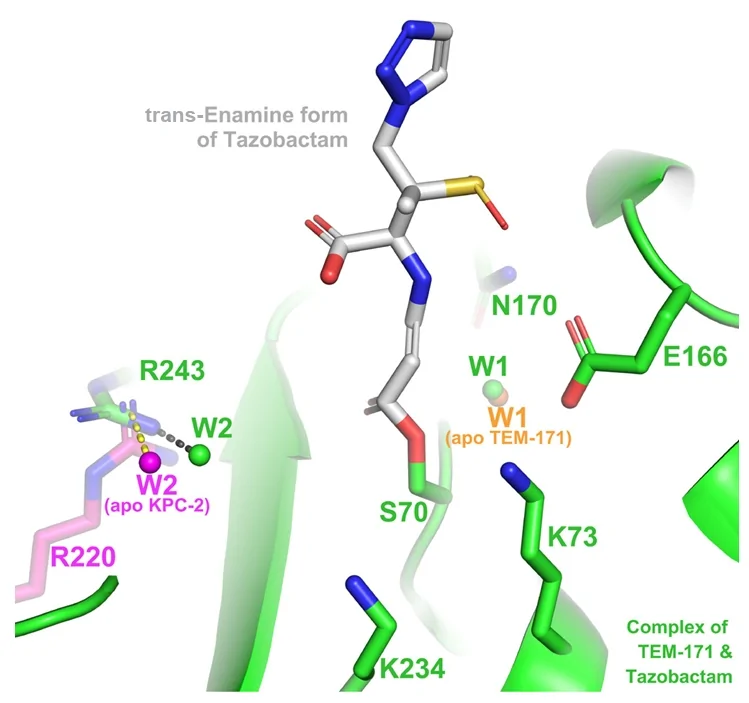
Comparison of deacylation water (W1) and second ring opening water (W2) in class A β-lactamases. TEM-171 apo form (PDB 7QLP, orange) was superposed with the TEM-171/Tazobactam (trans-enamine form) complex (PDB 7QOR, TEM-171 in green; Tazobactam in gray) and apo KPC-2 (PDB 5UL8, magenta). Deacylation water (W1) and the arginine-mediated water molecule (W2) are shown as spheres colored to match Cα atoms of each structure.

In detail, when the acyl-enzyme intermediate is formed, a water molecule coordinated by Arg244 for initial binding of carboxyl group mediates second ring opening of the β-lactam fused ring (oxazolidine or thiazolidine). This reaction leads to imine formation, which subsequently undergoes tautomerization to form a cis-enamine intermediate, followed by geometric isomerization to yield the trans-enamine intermediate (Fig. 8, Bonomo et al., 2001; Hinchliffe et al., 2022; Hugonnet and Blanchard, 2007; Lang et al., 2024). The imine intermediate is more susceptible to hydrolysis since its carbonyl carbon lacks conjugation, making it more electrophilic than that of the enamine. In contrast, the acylated carbon of the trans-enamine is stabilized through resonance delocalization (Fig. 8), which confers resistance to hydrolytic attack even though the deacylation water molecule is observed in the proper position, resulting in irreversible β-lactamase inhibition (Fig. 9, Kalp et al., 2009; Lang et al., 2024).

Compared with penam, penam sulfones have been reported to promote the second ring opening (Lang et al., 2022). However, the inhibitory activity also depends on the efficiency of trans-enamine formation within the active site. Consequently, no clear relationship has been established between the inhibitory activities of penam and penam sulfone among β-lactam-based BLIs (Payne et al., 1994).

β-Lactam–based inhibitors are ineffective against certain class A β-lactamases, such as KPC-2, which is the carbapenemase, because these enzymes lack the Arg244 (Arg243 in TEM-171) residue that is crucial for the second ring opening, which is essential for the stable inhibition. Although enzymes of this type possess an arginine residue at a nearby position (Arg220 in KPC-2), the coordinating water molecule is positioned farther from acylated β-lactam inhibitors, causing the second ring-opening step to be very slow or incomplete (Fig. 9, Papp-Wallace et al., 2012). Without this step, the inhibitor cannot convert into the stable trans-enamine intermediate, which leads to rapid deacylation of the acyl-enzyme complex and loss of inhibitory effect.

### The role of C2 substituents in stabilizing the trans-enamine intermediate

Sulbactam exhibits the weakest inhibitory activity and the narrowest inhibition spectrum among β-lactam-based inhibitors (Table 1, Kalp et al., 2009; Payne et al., 1994; Shapiro, 2017). Sulbactam possesses the simplest C2 substituent (Fig. 6), which limits formation of hydrogen bonding and hydrophobic interactions with the enzymes. In contrast, other β-lactam-based BLIs possess more complex C2 substituents that contribute to stabilizing the trans-enamine intermediate within the active site through such interactions. Previous studies have shown that Sulbactam forms a mixture of imine, cis-enamine, and trans-enamine intermediates in SHV-1 class A β-lactamase, whereas Tazobactam and Enmetazobactam intermediates predominantly exist as stable trans-enamine species (Kalp et al., 2009). It means that Sulbactam has relatively weak and narrow inhibitory activity because of its inability to effectively stabilize the trans-enamine intermediate.

### Another irreversible inhibition pathway of penam sulfones via dehydroalanine and lysinoalanine formation

**Fig. 10.**
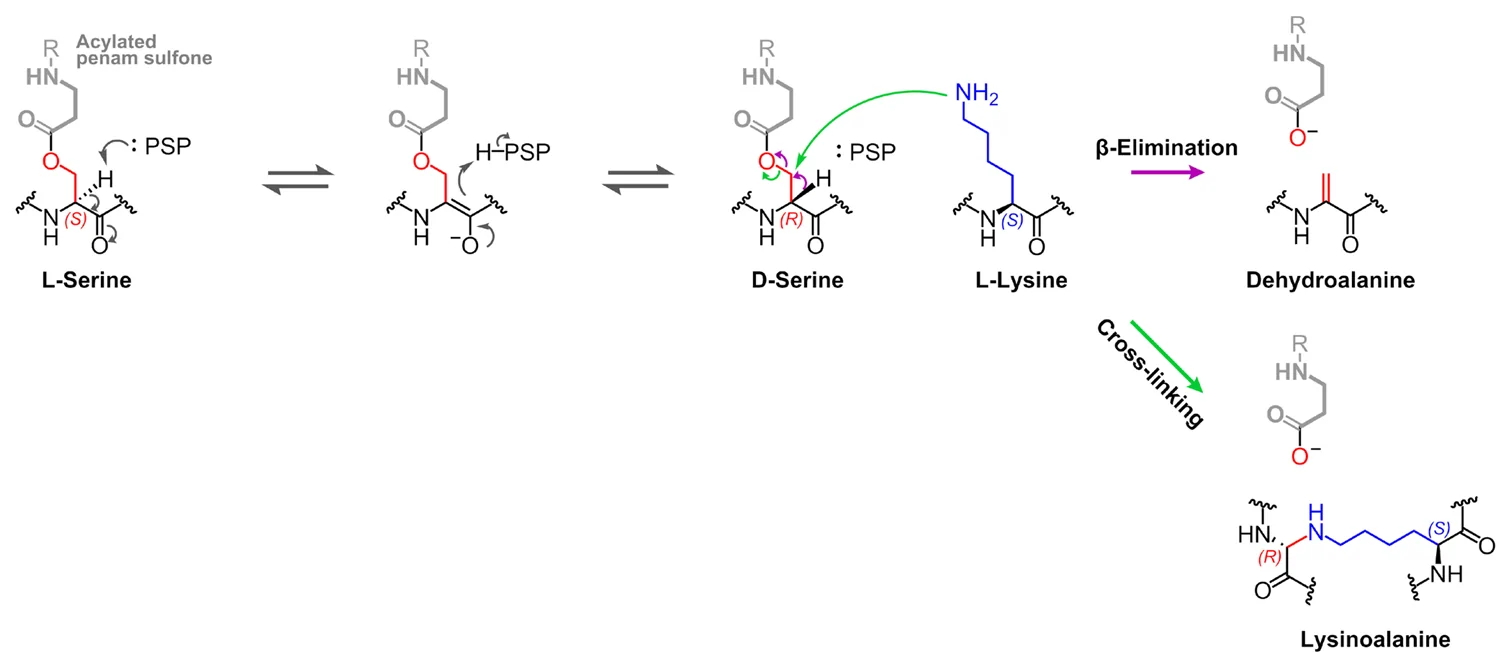
Irreversible inhibition mechanism of penam sulfone through dehydration of catalytic serine of SBLs. Serine and lysine side chains are shown in red and blue, respectively. L-Serine is converted to D-serine by an unidentified penam sulfone-derived product (PSP), which then undergoes β-elimination (purple arrows) to form dehydroalanine or cross-links with lysine to form lysinoalanine (green arrows), resulting in enzyme inactivation.

Penam sulfones inhibit β-lactamases not only through irreversible formation of a trans-enamine intermediate but also by modification of the nucleophilic serine residue and thereby permanently abolishing β-lactamase enzymatic activity (Fig. 10, Hinchliffe et al., 2022). The unidentified penam sulfone product (PSP) generated by β-lactamases is proposed to act as a strong base converting catalytic L-serine to D-serine. This conversion results in dehydroalanine by losing the hydroxyl group of D-serine (β-elimination). Some of these modified residues form a cross-link with a lysine residue, which is lysinoalanine (Fig. 10, Hinchliffe et al., 2022). This reaction has been observed only under high concentration of penam sulfone and long incubation times, and its occurrence under physiological conditions remains to be verified. Nevertheless, this finding suggests significant potential for advancing irreversible SBL inhibitors via a dehydroalanine-mediated pathway (Lang et al., 2022).

### Active site diversity limits β-lactam inhibitor efficacy in class C and D β-lactamases

β-Lactam-based BLIs effectively inhibit some class A β-lactamases, but exhibit reduced inhibitory activity against class C and class D β-lactamases because of distinct active sites. The active site of class C β-lactamases is composed of longer and more flexible loops than those of class A enzymes, making it difficult for β-lactam BLIs to form specific interactions (Fig. 4A and 4B). Additionally, the arginine residue in the carboxylate-binding subsite of class A enzymes is crucial for initial inhibitor binding, whereas the corresponding residue in class C enzymes is Asn346 (Fig. 4A and 4B). This difference reduces electrostatic interactions with the carboxylate group in BLIs. As a result, acylation occurs very slowly in class C β-lactamases (Monnaie and Frere, 1993). Moreover, even when an acyl-enzyme intermediate forms, the absence of Arg244, which additionally functions in the second ring opening in the carboxylate-binding subsite, prevents its conversion into the stable trans-enamine form leading to rapid hydrolysis (Fig. 8, Bonomo et al., 2001; Lobkovsky et al., 1993). Arg349 in the class C active site is positioned somewhat similarly to Arg244, but it lies too distant to establish a salt bridge with the carboxylate group or to mediate the second ring opening.

β-Lactam based inhibitors exhibit little to no efficacy against class D β-lactamases, likely due to their relatively hydrophobic active sites and the use of KCX residue as a general base (Fig. 3C, Mora-Ochomogo and Lohans, 2021). These structural differences may affect specific binding to β-lactam inhibitors or impede its conversion to the stable trans-enamine form.

## DBO-based BLIs

### DBO-based inhibitors: Avibactam to Durlobactam

Avibactam is the first non‑β‑lactam BLI, introducing the DBO scaffold with a carbonyl oxygen at C7, an amide group at C2 and a sulfate group at N6. Relebactam and Durlobactam were subsequently developed based on the Avibactam; Relebactam features an additional piperidine ring at the C2 amide, while Durlobactam introduces a C3–C4 double bond and a C3 methyl substituent (Fig. 6). These DBO-based BLIs show inhibitory activity against SBLs through mechanisms distinct from β-lactam based BLIs.

### Bifurcating deacylation pathway of DBO-based BLIs: Recyclization or desulfation

**Fig. 11.**
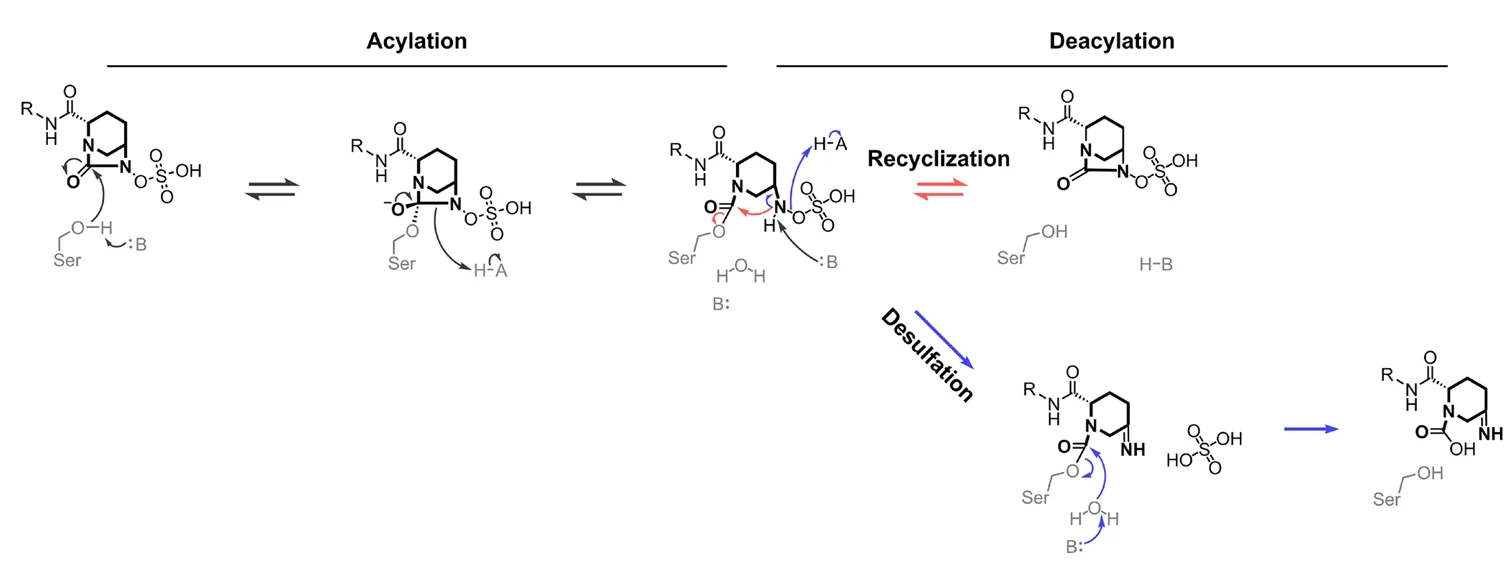
Two deacylation pathways of DBO-based BLIs by SBLs. DBO-based BLIs undergo deacylation after acyl-enzyme complex formation, either through recyclization to the intact form (red arrow) or via a desulfation pathway leading to an inactive form (blue arrow).

DBO-based inhibitors exhibit potent inhibitory activity not only against class A β-lactamases, which are targeted by β-lactam inhibitors, but also against class C β-lactamases and some class D β-lactamases, except that Relebactam does not inhibit class D (Table 1, Lizana et al., 2022; Tooke et al., 2019b). The acylation of DBO-based BLIs to catalytic serine of SBLs occurs in a manner similar to that of β-lactam-based BLIs. The deacylation, in contrast, proceeds via two distinct pathways: recyclization and desulfation (Fig. 11, Choi et al., 2016; Ehmann et al., 2013; Lang et al., 2021).

In the recyclization mechanism, unlike β-lactam inhibitors, acylated DBO inhibitors are not fragmented. Instead, they can regenerate their original structure via ring recyclization. Recyclization occurs through an intramolecular nucleophilic attack within the acyl-enzyme intermediate. The N6 is activated by a general base and attacks the carbonyl carbon, followed by proton transfer via a general acid, leading to deacylation (Figs. 6 and 11). As a result, the dissociated intact inhibitors allow for continual, repeated inhibition of SBLs (Choi et al., 2016; Ehmann et al., 2013; Lizana et al., 2022).

In the desulfation pathway, the general base assumed to be either the serine residue (Ser130 in KPC-2) or the deacylation water initiates desulfation, which leads to structural rearrangement into an imine form. Subsequently, the deacylation water attacks and cleaves the acyl-enzyme linkage, releasing the degraded inactive products (Fig. 11, Ehmann et al., 2013; Tooke et al., 2019b).

### Deacylation of DBO inhibitors: Recyclization dominance in class A and C β-lactamases and the KPC-2 desulfation exception

**Fig. 12.**
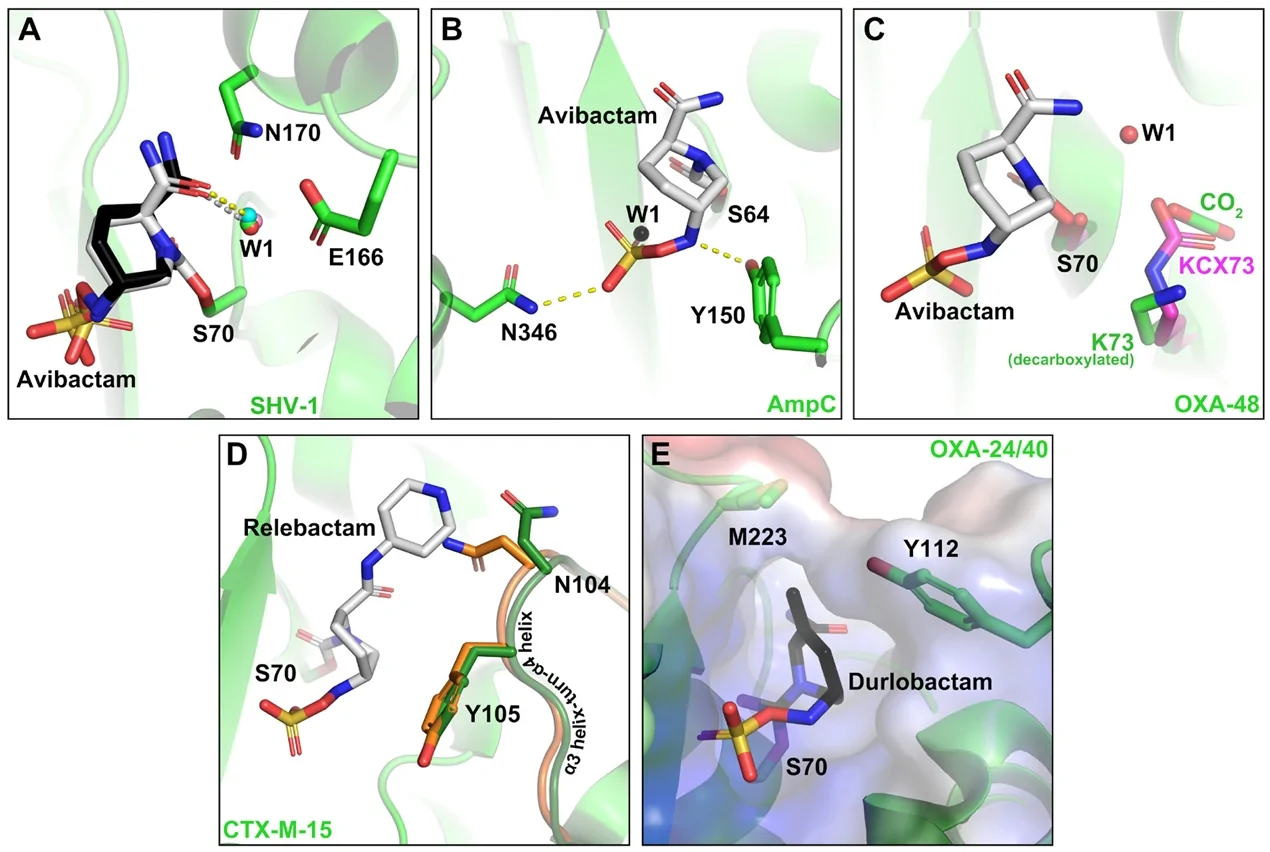
DBO-based BLI bound structures with SBLs. β-Lactamases are shown in cartoon representation, residues and inhibitors as sticks, and deacylation water (W1) as spheres colored to match Cα atoms of each structure. (A) Superposition of apo SHV-1 (PDB 4FH4, red) and SHV-1/Avibactam complex (PDB 4ZAM, SHV-1 in green; Avibactam in gray) with apo TEM-1 (PDB 5HVI, pink) and TEM-1/Avibactam complex (PDB 8DE0, TEM-1 in cyan; Avibactam in black). In both enzymes, the deacylation water (W1, sphere) retains in the Avibactam complex as in the apo structure. (B) Superposition of AmpC/Avibactam complex (PDB 4OOY, AmpC in green; Avibactam in gray) with apo AmpC (PDB 2BLS, black). The sulfate group of Avibactam aligns with the deacylation water (W1, black sphere) observed in the apo structure. (C) Superposition of apo OXA-48 (PDB 9H11, magenta) and OXA-48/Avibactam complex (PDB 4WMC, OXA-48 in green; Avibactam in gray), displaying the carboxylated state of the general base Lys73. Upon Avibactam binding, Lys73 becomes decarboxylated and CO₂ (green stick), presumed as the product. (D) Superposition of apo CTX-M-15 (PDB 4HBT, orange) and CTX-M-15/Relebactam complex (PDB 6QW8, CTX-M-15 in green; Relebactam in gray) demonstrates that upon Relebactam binding, Asn104 in the α3 helix-turn-α4 helix (bold color) moves outward toward the solvent to avoid steric clash. (E) Structure of OXA-24/40 bound to Durlobactam (PDB 6MPQ, OXA-24/40 in green; Durlobactam in black). Surface coloring represents negatively charged regions (red), positively charged regions (blue), and hydrophobic regions (white). Met223 and Tyr112 form a hydrophobic bridge creating a tunnel-like active site. C3 methyl group of Durlobactam engages in Met223 of the hydrophobic bridge.

DBO-based inhibitors extend their inhibitory spectrum to encompass both class A and class C β-lactamases by combining the prolonged active-site residence of the acyl-enzyme adduct, similar to that observed in β-lactam–based BLIs, with reversible deacylation through a recyclization pathway. In the acyl-enzyme complex formed between DBO inhibitors and class C β-lactamases, the sulfate group occupies the position of the deacylation water (Fig. 12B, Lahiri et al., 2014b; Usher et al., 1998), so the water-mediated desulfation reaction rarely occurs (Fig. 11, Usher et al., 1998). As a result, DBO-based inhibitors predominantly undergo recyclization and reversibly inhibit class C enzymes by regenerating intact inhibitors (Ehmann et al., 2013). In class A β-lactamases, the deacylation water molecule is positioned appropriately for catalysis (Fig. 12A, Ji et al., 2022; Krishnan et al., 2015; Lahiri et al., 2013). However, in the case of CTX-M-15, the catalytic base (Glu166) was found to be protonated, rendering it incapable of deprotonating the deacylation water (Lahiri et al., 2013). Molecular dynamics simulations revealed that the distance between the N6 atom and the carbonyl carbon of Avibactam is shorter than that between the deacylation water and the carbonyl carbon, indicating that the intramolecular nucleophilic attack is energetically more favorable (Choi et al., 2016). Therefore, despite the proper positioning of the deacylation water, the recyclization pathway is likely to be preferred to the desulfation pathway in class A β-lactamases.

**Fig. 13.**
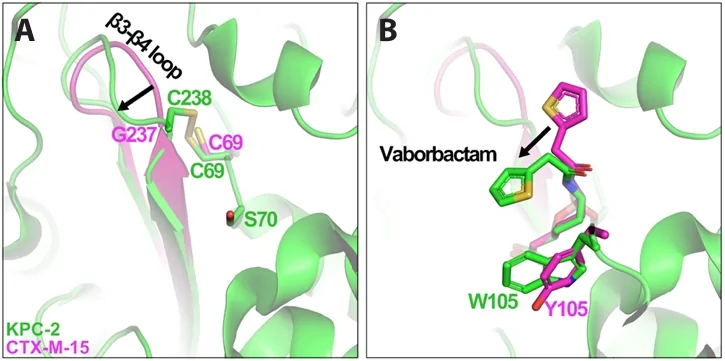
Comparison of the thiophene ring conformation of Vaborbactam between KPC-2 and CTX-M-15. Superposition of the KPC-2/Vaborbactam complex (PDB 6V7I, green) and the CTX-M-15/Vaborbactam complex (PDB 4XUZ, magenta) reveals that (A) a unique disulfide bond in KPC-2 (Cys69–Cys238) induces movement of the β3–β4 loop compared to CTX-M-15. (B) This loop shift in KPC-2 repositions the thiophene ring of Vaborbactam to interact hydrophobically with Trp105, adopting a U-shaped conformation (green stick), in contrast to the Z-shaped conformation observed in CTX-M-15 (magenta stick).

KPC-2 is an exceptional class A β-lactamase that exhibits a slow desulfation pathway, resulting in about 90% degradation of the inhibitor within 24 h (Krishnan et al., 2015). Although the exact structural basis of this process remains unclear, two possible general bases for desulfation have been proposed: the deacylation water molecule and Ser130 in the active site of KPC-2 (Fig. 3A). Each hypothesis requires the inhibitor to adopt a different conformation (Ehmann et al., 2013). When the deacylation water initiates desulfation, the N6 sulfate is assumed to invert toward the amide-binding subsite enabling nucleophilic attack (Figs. 4A and 6). This reaction may occur because KPC-2 possesses a disulfide bond adjacent to the active site that is absent in other class A enzymes (Fig. 13A). The disulfide bond shifts the main chain carbonyl oxygen of Thr237 by about 1.5 Å, allowing it to interact with the desulfated imine intermediate and potentially stabilizing it, which may promote desulfation during deacylation (Alsenani et al., 2023). In the second hypothesis, conversely, the N6 sulfate may orient toward the carboxylate-binding subsite for Ser130 acting as the base that triggers desulfation (Figs. 4A and 6). Ser130 is conserved in all class A β-lactamases, but the unique microenvironment of the active site in KPC-2 can create a distinct spatial arrangement between the DBO inhibitor and Ser130 that may facilitate the nucleophilic attack required for desulfation (Krishnan et al., 2015).

### Distinct irreversible inhibition pathway of class D β-lactamases: DBO-mediated KCX decarboxylation

In contrast, DBO-based BLIs show “almost irreversible” inhibition against class D β-lactamases. Based on reported investigations of their acyl-enzyme complexes using ^19^F NMR and X-ray crystallography, the KCX residue, the general base of class D β-lactamases involved in deacylation, is observed in an inactive decarboxylated form. According to the prevailing hypothesis, this modification results from changes in the KCX microenvironment induced by the acylated DBO inhibitor, which shifts its pKa and promotes decarboxylation, resulting in irreversible inhibition (Fig. 12C, Barnes et al., 2019; Ehmann et al., 2013; Hoff et al., 2025; Lahiri et al., 2015).

### Influence of substituents of DBO-based BLIs on their inhibitory spectrum: Relebactam vs. Durlobactam

Relebactam and Durlobactam, derivatives of the basic Avibactam scaffold, exhibit distinct inhibitory efficacy and spectra due to differences in their substituents (Fig. 6, Table 1). Relebactam with a bulky piperidine ring at C2 is the only DBO inhibitor that does not inhibit class D β-lactamases (Fig. 6, Table 1, Papp-Wallace et al., 2018). The shallow or tunnel-like configuration of their active sites may restrict access of the bulky molecule (Fig. 5). In addition, Relebactam shows weaker inhibitory activity against class A β-lactamases compared to Avibactam, as the piperidine ring induces steric hindrance at the α3 helix–turn–α4 helix in the L1 loop of these enzymes (Figs. 4A and 12D, Tooke et al., 2019b). On the other hand, Relebactam resists efflux pump–mediated drug export due to its piperidine ring (Young et al., 2019), thereby compensating for the reduced enzyme inhibition. As a result, both Relebactam and Avibactam similarly restore β-lactam antibiotic efficacy against class A β-lactamase-producing bacteria (Tooke et al., 2019b).

Durlobactam exhibits a broader inhibitory spectrum against class D β-lactamases and enhanced inhibitory activity compared to Avibactam (Table 1). The double bond between C3 and C4 enhances the reactivity of Durlobactam by increasing ring strain and electrophilicity of the carbonyl carbon (Fig. 6), lowering the activation energy barrier of the nucleophilic attack by the catalytic serine of SBLs and consequently improving inhibitory activity (Lizana et al., 2022; Shapiro et al., 2021). A C3 methyl group of Durlobactam was introduced to extend its inhibitory spectrum toward class D β-lactamases featuring a hydrophobic bridge (Phe110–Met221 in OXA-23 or Tyr112–Met223 in OXA-24, Figs. 5 and 12E) that are poorly inhibited by Avibactam (Durand-Reville et al., 2017). The C3 methyl group of Durlobactam forms hydrophobic interactions with the methionine residue within the bridge, stabilizing the binding conformation, thus allowing Durlobactam to overcome the steric constraints that limit other DBO inhibitors against class D β-lactamases containing hydrophobic bridges (Fig. 12E, Shapiro et al., 2021).

## Boronic Acid-based BLIs

### The first and unique boronic acid β-lactamase inhibitor: Vaborbactam

Vaborbactam is the first and only FDA-approved BLI with a boronic acid core, discovered through computer-based drug design strategies to inhibit SBLs including KPC-type carbapenemases (Hecker et al., 2015). Structurally, Vaborbactam contains a six-membered cyclic boronic acid ring in which the boron atom is coordinated to two oxygen atoms—one incorporated into the ring as an endocyclic ester and the other existing as an exocyclic hydroxyl group. The molecule is further substituted with a 2-thiophene ring via an acetamido at the C3 position and an acetic acid moiety at C6 (Fig. 6).

### Boron hybridization dynamics underlying broad β-lactamase inhibition

The inhibition mechanism of boronic acid-based inhibitors differs fundamentally from that of β-lactam– or DBO-based BLIs. Boron exhibits a unique structural plasticity, interconverting between trigonal planar sp^2^ hybridization with a vacant *p*-orbital and tetrahedral sp^3^ geometry (Diaz and Yudin, 2017). The sp^2^-hybridized boron atom structurally and electronically resembles the carbonyl carbon present in the β-lactam ring, allowing it to be recognized by β-lactamases as a “substrate-like” species and undergo nucleophilic attack, resulting in a transition to the sp^3^-hybridized state (Fig. 7). The sp^3^ geometry mimics the common tetrahedral intermediate observed in both acylated and metal-coordinated β-lactam substrates, without β-lactam ring opening, in SBLs and MBLs, respectively (Figs. 2 and 7, Krajnc et al., 2019). Therefore, boronic acid serves as a highly promising core structure for pan-class BLIs.

### Vaborbactam-KPC interactions mediated by active-site disulfide bond and thiophene packing

Although Vaborbactam, a boronic acid-based BLI, has demonstrated potent inhibitory activity against KPC-type class A carbapenemases, its inhibition spectrum is relatively narrow, mainly limited to class A and class C SBLs (Table 1, Tsivkovski and Lomovskaya, 2020). Vaborbactam almost irreversibly inhibits KPC enzymes which have a unique structural feature among class A SBLs—the disulfide bond (Cys69–Cys238) located near the active site (Fig. 13A). This bond modulates the positioning of the β3-β4 loop, which induces rotation of the thiophene ring of Vaborbactam and a compact interaction with Trp105, stabilizing its residence in the active site (Fig. 13B, Pemberton et al., 2020). Vaborbactam dissociates slowly from KPC enzymes without structural changes, allowing the released molecule to remain intact and rebind to KPC enzymes for inhibition (Tsivkovski and Lomovskaya, 2020).

### Influence of conformational plasticity and the dynamics of the acylated B–O bond on Vaborbactam efficiency

The inhibitory activity of Vaborbactam across both class A and class C β-lactamases can be attributed to its conformational flexibility. Its non-resonating cyclic boron ring, the acetic acid substituent that extends the molecular geometry, and the thiophene ring containing a spacer carbon collectively enable the molecule to adopt diverse binding conformations suited to different enzyme active sites. Crystallographic analyses have revealed distinct binding modes—such as a U-shaped binding mode in KPC-2, a Z-shaped conformation in CTX-M-15 (Fig. 13B), and a more extended configuration in AmpC—demonstrating its remarkable structural adaptability (Hecker et al., 2015; Lomovskaya et al., 2017).

By contrast, such conformational plasticity may interfere with the stable association required for effective inhibition in certain β-lactamases including class D SBLs and MBLs, potentially narrowing down its inhibition spectrum (Table 1). The efficacy of the B-O bond cleavage between boronic acids and the catalytic serine may also influence the spectrum against SBLs (Tooke et al., 2020). However, the molecular mechanism of this cleavage remains unclear. Investigating the correlation between the inhibition spectrum of Vaborbactam and its structure–activity relationship might provide valuable insights for designing inhibitors that can inhibit both SBLs and MBLs and stably reside within the active site once forming tetrahedral intermediates.

## Expanding the Therapeutic Potential of BLIs through PBP Inhibition

Beyond β-lactamase inhibition, some FDA-approved BLIs exhibit intrinsic antibacterial activity through inhibition of PBPs (Asli et al., 2016; Finlay et al., 2003; Kumar et al., 2021; Moya et al., 2017; Papp-Wallace et al., 2023), which are evolutionarily related to SBLs as members of the active site serine penicillin-recognizing enzymes (ASPRE) family. This dual activity underlies the FDA approval of combinations such as Sulbactam and Durlobactam for antibacterial therapy without any β-lactam antibiotics (Table 1). While this review does not address it in detail, continued research on the mechanisms and structural basis of the expanding PBP inhibitory activity of BLIs is essential for further improving their therapeutic efficacy.

## Clinical Emergence of BLI-Resistant Variants

**Fig. 14.**
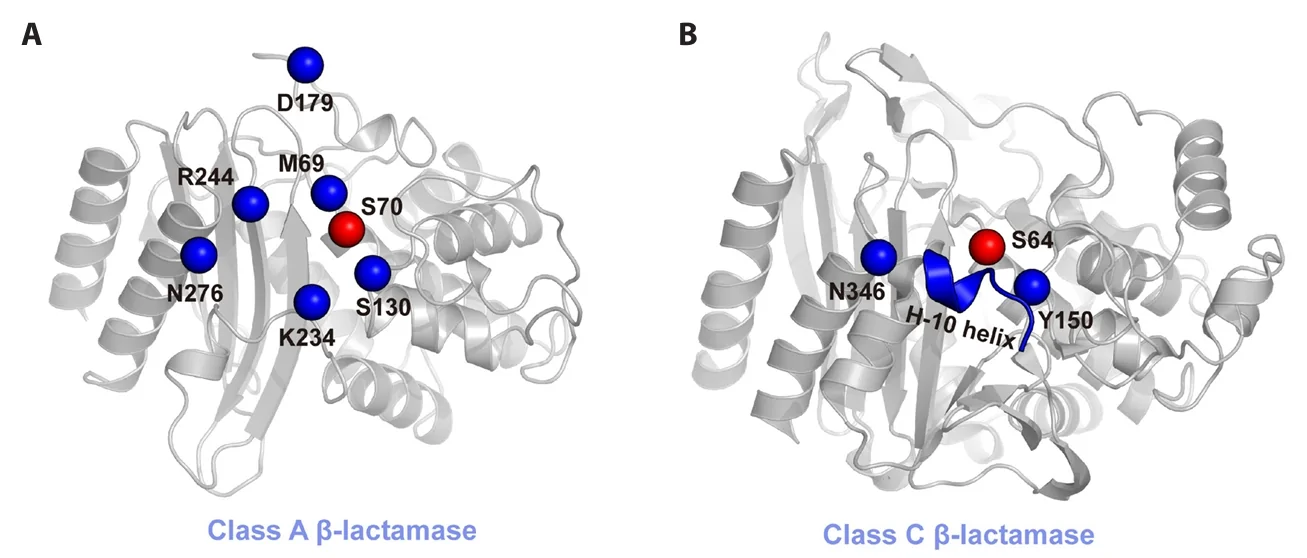
The distribution of inhibitor-resistant (IR) mutation sites in class A and class C β-lactamases. Overall structures are shown in gray; (A) apo TEM-1 (PDB 1BTL) and (B) apo AmpC EC-1 (PDB 1KE4). The Cα atoms are shown as spheres, with the catalytic serine in red, which is not associated with IR mutations. Residues with clinically observed IR mutations are shown in blue.

The emergence of naturally and clinically occurring inhibitor-resistant β-lactamase variants (IR variants) has led to the failure of β-lactam/BLI combinations in clinical therapy (Fig. 14, Papp-Wallace, 2019). The IR variants carry substitutions that convert previously inhibitor-susceptible β-lactamases into inhibitor-resistant forms. Thus, interpretation of IR variants in the context of how residue-level modifications structurally influence the inhibitory pathway could provide the foundation for structure-based inhibitor design capable of overcoming these resistance mechanisms.

## Structural Mechanisms of Resistance to β-Lactam-Based Inhibitors

### Mutation-driven loss of the carboxylate-binding subsite in class A β-lactamases

Among class A β-lactamases, TEM- and SHV-type are highly inhibited by β-lactam–based BLIs, which critically depends on a well-organized carboxylate-binding subsite. Arg244 in this site plays a dual role in electrostatically anchoring the C3 carboxylate of the inhibitors (Figs. 6 and 14A) and aligning deprotonating water molecule that mediates second-ring opening leading to trans-enamine formation for irreversible inhibition (Figs. 8 and 9, Drawz and Bonomo, 2010; Imtiaz et al., 1993). The R244S/H/C substitutions in TEM-type disrupt this electrostatic network and inhibit the second ring opening, causing a 10–20-fold increase in *K*_i_ values (Canica et al., 1998; Canton et al., 2008; Giakkoupi et al., 1998b; Marciano et al., 2009).

**Fig. 15.**
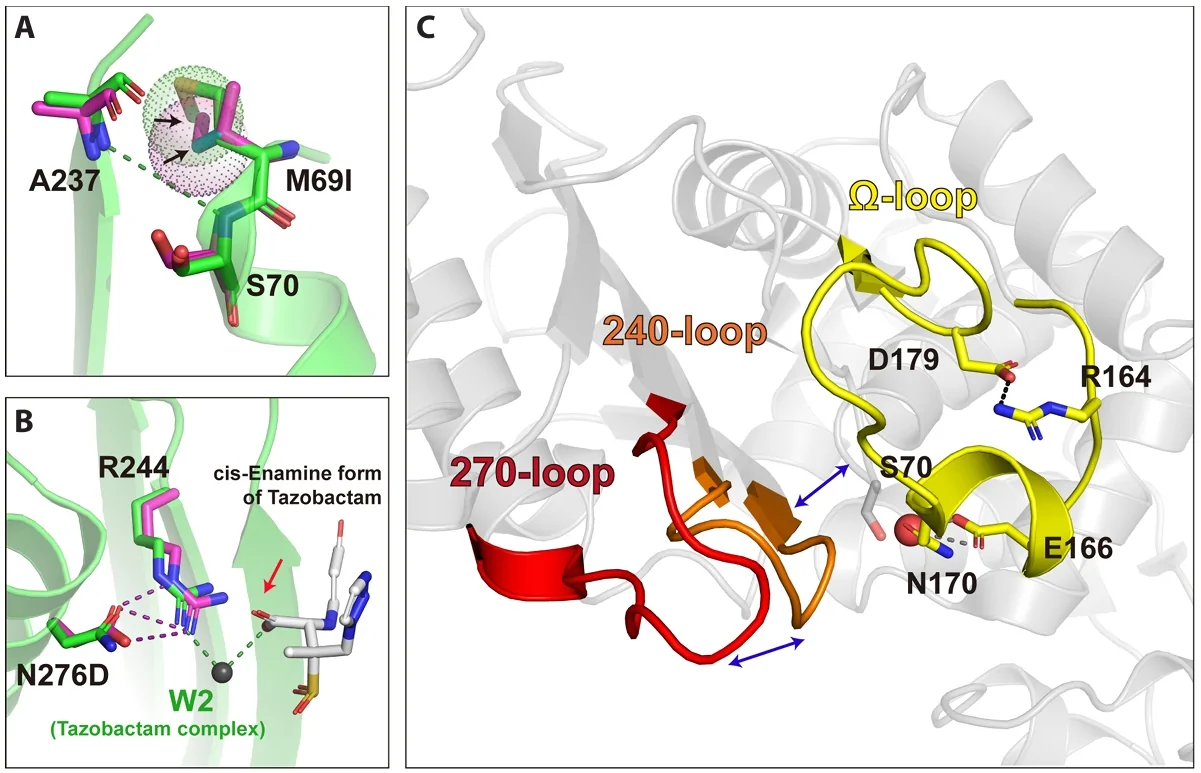
Structural features of inhibitor-resistant (IR) variants in class A β-lactamases. (A) Superposition of TEM-1 wildtype (PDB 1BTL, green) and TEM-1 M69I mutant (PDB 1LI0, magenta) structures. Residues forming the oxyanion hole are shown as sticks. The side-chain volume of residue 69, forming the back-wall of the oxyanion hole, is represented by dots. The black arrows mark atoms showing a spatial gap between the wildtype and mutant structures. (B) Structural comparison of TEM-1/Tazobactam (cis-enamine form) complex (PDB 7QNK, TEM-1 in green; Tazobactam in gray) and TEM-1 N276D mutant (PDB 1CK3, magenta). Residues N276D, Arg244, and Tazobactam are shown as sticks. The water molecule involved in the second ring opening is shown as a gray sphere (W2), which is not observed in TEM-1 N276D. The carboxylate group of Tazobactam is indicated by a red arrow. The electrostatic interaction between N276D and Arg244 is represented by purple dashed lines. Hydrogen bonds connecting Arg244, the water molecule, and the C3 carboxylate group of Tazobactam are represented by green dashed lines. (C) The inter- and intra-loop network observed in the KPC-2 (PDB 5UL8). The Ω-loop, 240-loop, and 270-loop are colored yellow, orange, and red, respectively. Inter-loop interactions are represented as blue arrows. The residues involving intra-loop interaction and coordination of the deacylation water (red sphere) are shown as sticks.

The spatial orientation of Arg244 for its dual role is maintained through a hydrogen bond with Asn276. The N276D variant neutralizes the positive charge of Arg244, reducing carboxylate affinity and misaligning the deprotonating water molecule required for second-ring opening, which in turn favors deacylation over conversion to the trans-enamine intermediate (Figs. 8 and 15B, Canica et al., 1998; Drawz et al., 2009; Papp-Wallace et al., 2012; Swaren et al., 1999).

### Mutation-associated destabilization of the oxyanion hole in class A β-lactamases

Although all BLIs utilize the oxyanion hole to stabilize their tetrahedral intermediates, β-lactam–based inhibitors are particularly dependent on the oxyanion hole. The β-lactam ring has a single nitrogen and a highly strained four-membered ring, making the oxyanion of its tetrahedral intermediate more negative than that of the DBO ring with two electron-withdrawing nitrogen atoms and less ring strain (Fig. 6, Coleman, 2011; Ehmann et al., 2012). In naturally occurring β-lactam–based BLI-resistant variants of TEM and SHV, oxyanion hole–associated substitutions at Met69—such as M69I, M69L, or M69V—have been identified. In class A β-lactamases, the oxyanion hole is universally formed by the backbone amide group of Ser70 and Ala237 (Fig. 15A). The side chain of Met69 shapes the back wall of the oxyanion hole in TEM and SHV (Fig. 15A). Thus, replacement of Met69 with more hydrophobic and branched residues perturbs the oxyanion hole by imposing steric constraints on the back-wall, increasing the resistance to β-lactam BLIs (Fig. 15A, Bonomo et al., 1995; Chaibi et al., 1998; Giakkoupi et al., 1998a).

### Mutations in the Lys73–Ser130–Lys234 network impair the acylation step in class A β-lactamases

In class A β-lactamases, Ser130 serves as the central mediator within the Lys73–Ser130–Lys234 proton-shuttle network (Fig. 3A). This network activates catalytic Ser70 by deprotonation and transfers the proton to the amide nitrogen of β-lactam ring, promoting the ring opening and the conversion of the tetrahedral intermediate into the acyl-enzyme intermediate (Fig. 7, Soeung et al., 2020; Sun et al., 2004). Substitution of Ser130 with glycine or threonine (S130G/T) perturbs the local electrostatic environment for this proton shuttle, slowing the entire acylation step (Helfand et al., 2003; Thomas et al., 2005).

Within this network, the lysine residues play a critical electrostatic role in lowering the *pK*_a_ and maintaining the proper orientation of Ser130 (Fig. 3A). When Lys234 is replaced with arginine, which exhibits electron delocalization within the guanidinium group, the *pK*_a_-lowering effect is diminished. Structurally, Ser130 redirects its side chain toward K234R, forming a hydrogen bond with a guanidinium nitrogen of arginine (Soeung et al., 2020). This reorientation causes the side chain of Ser130 to shift away from both the β-lactam amide nitrogen and Lys73, thereby disrupting the Lys73–Ser130–Lys234 proton-transfer alignment and reducing the acylation efficiency (Canton et al., 2008; Elings et al., 2021; Soeung et al., 2020).

## Structural Mechanisms of Resistance to DBO-Based Inhibitors

### The Ω-loop mutation–induced inter-loop disorganization weakens inhibitor binding in class A β-lactamases

The KPC-type β-lactamases, the most prevalent and clinically significant class A carbapenemases, harbors variants that confer resistance to DBO-based inhibitors. It is predominantly associated with the amide-binding subsite (Fig. 4A). Within this subsite, the Ω-loop provides interactions with the amide bond of Avibactam, including van der Waals contacts and a hydrogen bond with the deacylation water coordinated by Glu166 and Asn170 in the Ω-loop (Fig. 12A, Alsenani et al., 2022, 2023). Thus, the proper conformation of the Ω-loop to maintain these interactions is critical for the tight binding of Avibactam.

Asp179 contributes to stable conformation and rigidity of the Ω-loop through an intra-loop salt bridge with Arg164 (Fig. 15C). The D179Y substitution with relatively neutralized charge and increased size compared to aspartate disrupts this salt bridge and introduces steric hindrance with residues in the Ω-loop. As a result, the Ω-loop becomes fully disordered impairing the ability to interact with Avibactam, thereby decreasing the acylation efficiency (*k*_2_/*K_i_*, Alsenani et al., 2022; Compain and Arthur, 2017). Although the D179Y variant perturbs the deacylation water positioning, it retains catalytic activity against substrates (e.g., ceftazidime; Alsenani et al., 2022). The increased flexibility of the Ω-loop allows better accommodation of bulky substituents attached to the amide bond of β-lactam antibiotics that cannot be accommodated in the rigid Ω-loop of wild-type. This compensates for the slowing of the deacylation step caused by altered positioning of the deacylation water.

The conformation of Ω-loop is further influenced by inter-loop interactions with the adjacent 240- and 270-loops through an intricate packing network (Fig. 15C). Mutations within these regions modulate Ω-loop architecture and inhibitor susceptibility. A 15-amino-acid duplication or a DDK insertion within the 270-loop induces its disordering. These structural changes disorganize the conformations of the adjacent 240- and Ω-loops in a domino-like effect and alter the spatial arrangement of the active site for Avibactam binding, resulting in diminished acylation efficiency (Sun et al., 2024).

### Trade-off between catalytic activity and inhibitor resistance in Tyr150 variants of class C β-lactamases

The DBO core specifically interacts with a highly conserved region of class C β-lactamases, which is essential for β-lactam catalysis (Lahiri et al., 2014b). It means that the enzymes must alter residues related to catalytic efficiency to develop resistance to DBO-based BLIs. Tyr150 is a strictly conserved catalytic residue across class C β-lactamases that generally functions as a general acid/base that activates the catalytic serine for nucleophilic attack (Figs. 3B and 14B, Lahiri et al., 2014b). The Y150C/S substitution shortens the side chain of the general base residue, thereby increasing the distance from the catalytic serine (Lahiri et al., 2014b; Philippon et al., 2022; Russ et al., 2020). Additionally, Tyr150 also forms a hydrogen bond with N6 atom of Avibactam (Fig. 12B), which is not observed in the interactions between β-lactam substrates and class C β-lactamases (Das and Nair, 2018; Lahiri et al., 2014b). The Y150C/S substitution destabilizes Avibactam binding due to the loss of this hydrogen bond within the active site. Together, this alteration produces a drastic reduction in inhibition efficiency (*K*_i_) toward Avibactam, accompanied by a sacrifice in catalytic efficiency (decreasing *k*_cat_/*K*_M_) toward substrates (Lahiri et al., 2014b; Philippon et al., 2022).

### The carboxylate-binding subsite variants impair sulfate anchoring and drive DBO-based BLIs resistance in class C β-lactamases

The carboxylate-binding subsite of class C β-lactamases is a mutational hotspot (Figs. 4B and 14B). Alterations in this region can affect accommodation of both bulkier β-lactam substrates and DBO-based BLIs. Asn346, conserved in over 95% of class C β-lactamases and composing the carboxylate-binding subsite (Fig. 4B), forms a hydrogen bond with the sulfate group of Avibactam, thereby anchoring and stabilizing the inhibitor within the active site (Fig. 12B, Lahiri et al., 2014b; Philippon et al., 2022). The N346Y substitution identified in clinical isolates introduces steric hindrance from the bulky tyrosine side chain, markedly reducing the Avibactam affinity and acylation efficiency (*k*_2_/*K*_i_, Lahiri et al., 2014b). β-Lactam substrates also position their carboxylate or sulfonyl groups near Asn346, but its position is slightly farther than Avibactam, thereby the effect of N346Y is spatially less constrained than that with Avibactam and largely preserving β-lactam hydrolysis (Compain et al., 2020). Thus, unlike Y150C/S, the N346Y variant represents an evolutionarily permissible resistance pathway with minimal functional trade-off (Compain et al., 2020; Lahiri et al., 2014b).

The H-10 helix (residues 289–296), located within the R2 loop (residues 289–307), forms the bottom of the carboxylate-binding subsite as a hydrophobic surface that mediates the proper positioning of the sulfate group in Avibactam for optimal interaction with Asn346 (Figs. 4B and 14B, Lahiri et al., 2014a). Deletions within this region, such as the six–amino acid deletion (SKVALA 289–294 in AmpC) or the two–amino acid deletion (A292–L293 in AmpC) variants, lose this hydrophobic supporting surface, thereby leading to a subtle rearrangement of the sulfate anchoring geometry (Philippon et al., 2022; Shields et al., 2020). Thus, inhibitor binding is weakened but not totally abolished (Lahiri et al., 2014a). Such deletions are also known to enable adaptive expansion of the carboxylate-binding subsite to easily accommodate larger substrates (Jeong et al., 2021; Shields et al., 2020).

## Vaborbactam Resistance Arises without Enzyme Modification

Unlike β-lactam or DBO inhibitors, clinical and experimental data show that resistance to Vaborbactam usually arises through non-enzymatic mechanisms, such as porin loss (e.g., OmpK35/OmpK36) or amplification of the *bla*KPC (Dulyayangkul et al., 2020; Sun et al., 2017). Mutations within the β-lactamase itself are extremely rare and are not considered a major contributor to clinical resistance.

## Discussion

FDA-approved BLIs share three common structural characteristics. The first is an electron-deficient atom; the second is an anionic functional group such as carboxylate or sulfate; the third is an amide bond including an enamine in the case of β-lactam–based inhibitors (Figs. 6 and 8). These structural motifs are also found in β-lactam antibiotics, the primary substrates of β-lactamases (Fig. 1). Thus, BLIs could mimic the enzyme’s natural catalytic intermediates during β-lactam hydrolysis. Specifically, the first feature facilitates the tetrahedral intermediate or acyl-enzyme complex during acylation in SBLs (Fig. 7). The second and third features interact with the conserved carboxylate-binding subsite and the amide-binding subsite within the SBL active site (Fig. 4). While this substrate-mimicking strategy enables effective inhibition, it also involves intrinsic limitations.

β-Lactam and DBO inhibitors which are designed based on the substrate catalytic mechanism of SBLs paradoxically render the inhibitors susceptible to recognition as substrates, leading to hydrolysis and inactivation. In addition, these inhibitors generally lack activity against MBLs (Table 1). In contrast, boronic acid–based inhibitors mimicking the common tetrahedral intermediate of SBLs and MBLs are not likely to be recognized as substrates and are expected to show inhibitory activity against MBLs. However, the boron atom may also bind to other serine- or metallo-hydrolases, raising the possibility of off-target effects and safety concerns.

Considering these aspects, new BLI development requires two complementary approaches. The first approach is the optimization of established core scaffolds. For example, modifications may increase the chemical stability of the trans-enamine formed after acylation in β-lactam-based BLIs (Fig. 8) or adjust recyclization pathways in DBO-based BLIs to avoid hydrolysis (Fig. 11). Additional substituents can enhance hydrogen bonding or other interactions, improving adaptability to diverse active-site environments. Boronic acid derivatives could offer a potential route to broader inhibition as they mimic the common tetrahedral intermediate of SBLs and MBLs (Figs. 2 and 7). An example is Taniborbactam, currently in phase 3 clinical trials, which inhibits both SBLs and MBLs by incorporating a bicyclic structure into the boronic acid scaffold (Liu et al., 2020). This illustrates the feasibility of scaffold refinement in broadening the inhibitory spectrum. This strategy could be supported by existing safety and pharmacological data, providing higher clinical applicability in the short term as part of resistance management.

The second approach is the identification of entirely new scaffolds distinct from current BLIs. Possible strategies include screening natural or synthetic small molecules that bind to β-lactamases, repositioning approved drugs, or isolating small proteins with β-lactamase inhibitory activity. This line of research has produced several preliminary findings. Fragment-based discovery has identified dipicolinic acid with activity against MBLs (Chen et al., 2017); nucleotides, which are natural products, have been reported to show inhibitory effects (Kim et al., 2017; Na et al., 2017, 2018); the antifungal agent Tavaborole has demonstrated BLI activity (Zhang et al., 2025); and β-lactamase inhibitory proteins have been reported (Strynadka et al., 1994). However, none of these candidates has yet progressed to FDA approval. Although still in early stages, these studies indicate new structural possibilities that could be important for long-term strategies to combat antibiotic resistance.

Future BLI development will likely require a balanced approach that combines scaffold optimization with the discovery of new structural motifs. The structural and mechanistic analyses summarized here may support the rational design of next-generation inhibitors and the exploration of innovative scaffolds.

## Figures and Tables

**Table 1.** FDA-approved BLIs: formulation and inhibitory spectrum

Core structure	Inhibitor	Partner β-lactam	Formulation (Year of FDA approval)	Inhibition profile of the inhibitor^†^								
				Class A				Class C		Class D		Class B
				TEM	SHV	CTX-M	KPC	AmpC	CMY	OXA-23	OXA-48	MBL^‡^
β-Lactam	Clavulanic acid	Amoxicillin	Augmentin (1984)	O	O	O	X	X	X	X	X	X
	Sulbactam	Ampicillin	Unasyn (1986)	O	△	△	X	X	X	X	X	X
	Tazobactam	Piperacillin	Zosyn (1993)	O	O	O	X	X	X	X	X	X
		Ceftolozane	Zerbaxa (2014)									
	Enmetazobactam	Cefepime	Exblifep (2024)	O	O	O	X	X	X	X	X	X
DBO^*^	Avibactam	Ceftazidime	Avycaz (2015)	O	O	O	O	O	O	X	O	X
		Aztreonam	Emblaveo (2025)									
	Relebactam	Imipenem and cilastatin	Recarbrio (2019)	O	O	O	O	O	O	X	X	X
	Durlobactam	Sulbactam	Xacduro (2023)	O	O	O	O	O	O	△	O	X
Boronic acid	Vaborbactam	Meropenem	Vabomere(2017)	O	O	O	O	O	O	X	X	X

† (Naas et al., 2017).

‡ metallo β-lactamase.

* diazabicyclooctane.

O: potent inhibition; △: moderate inhibition; X: no inhibitory activity.
